# Supplementary material for: Potential dissemination of IncHI2/IncHI2A plasmids carrying mcr-9.4 complex transposon in chicken-derived Enterobacter hormaechei
Source: Microbiol Spectr. 2026 Mar 16;14(4):e01979-25. doi: 10.1128/spectrum.01979-25 (PMC13055346; doi:10.1128/spectrum.01979-25)
Supplement: Supplemental material — Files S1 to S6. [file spectrum.01979-25-s0001.docx]

S 1 Concentration of antimicrobic

| **Antimicrobic** | **Code** | **Concentration(µg/mL)** |
| --- | --- | --- |
| Ampicillin | AMP | 4,8,32 |
| Amoxicillin/ClavulanicAcid | AMC | 4/2,16/8,32/16 |
| Ticarcillin/ClavulanicAcid | TEC | 8/2,32/2,64/2 |
| Cefalexin | CN | 8,32,64 |
| Cefalotin | CF | 2,8,32 |
| Cefoperazone | CFP | 4,8,32 |
| Ceftiofur | CFT | 1,2 |
| Cefquinome | CEQ | 0.5,1.5,4 |
| Imipenem | IPM | 1,2,6,12 |
| Gentamicin | GM | 4,16,32 |
| Neomycin | N | 8,16,64 |
| Flumequine | UB | 2,4,8 |
| Enrofloxacin | ENR | 0.25,1,4 |
| Marbofloxacin | MRB | 1,2 |
| Tetracycline | TE | 2,4,8 |
| PolymyxinB | PB | 0.25,1,4,16 |

S 2 Prediction of pGS32-1 replicon


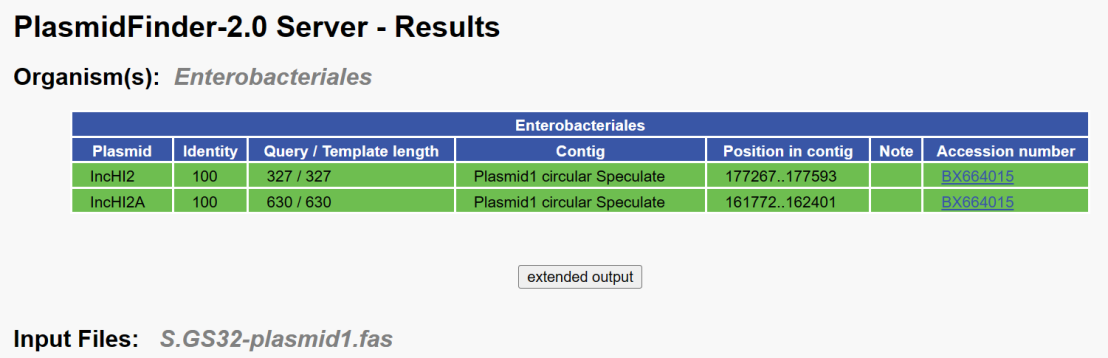


S 3 VRprofile2 predictions of pGS32-1

| MGE  Type | MGE Details | Position | Length | Antibiotic_Resistance_Genes | Drug_Class | Drugs |
| --- | --- | --- | --- | --- | --- | --- |
| Conjugative plasmid | - | 1..255043 | 255043 | mcr-9 / sul2 / floR / qnrS1 / qepA1 / aph(3')-Ia / aadA2 / tet(D) / mph(A) | Polymyxin / Sulphonamide / Phenicol / Fluoroquinolone / Fluoroquinolone / Aminoglycoside / Aminoglycoside / Tetracycline / Macrolide | Colistin / Sulfamethoxazole / Chloramphenicol, Florfenicol / Ciprofloxacin / Ciprofloxacin / Neomycin, Kanamycin, Lividomycin, Paromomycin, Ribostamycin / Spectinomycin, Streptomycin / Doxycycline, Tetracycline / Erythromycin, Azithromycin, Spiramycin, Telithromycin |
| IScluster/Tn | IS903B / ISKpn14 / IS1R | 541..6864 | 6323 | mcr-9 | Polymyxin | Colistin |
| IS/Tn | IS903B | 13086..14009 | 923 | - | - | - |
| IScluster/Tn | ISKpn19 / ISEc59 / ISRsp12 / ISAba33 / ISVsa3 / IS1006 / ISKpn19 / TnpR_TnMex22 / ISEae2 / IS26 / IS26 / IS15DI / TnpR_TnAs2 / TnpA_TnAs2 / IS4321R | 20482..57699 | 37217 | sul2 / floR / qnrS1 / qepA1 / aph(3')-Ia / aadA2 | Sulphonamide / Phenicol / Fluoroquinolone / Fluoroquinolone / Aminoglycoside / Aminoglycoside | Sulfamethoxazole / Chloramphenicol, Florfenicol / Ciprofloxacin / Ciprofloxacin / Neomycin, Kanamycin, Lividomycin, Paromomycin, Ribostamycin / Spectinomycin,Streptomycin |
| IScluster/Tn | IS26 / IS26 / IS6100 / IS4321R | 64903..77048 | 12145 | tet(D) / mph(A) | Tetracycline / Macrolide | Doxycycline, Tetracycline / Erythromycin, Azithromycin, Spiramycin, Telithromycin |
| IS/Tn | IS903B | 139226..140194 | 968 | - | - | - |
| IScluster/Tn | ISKpn14 / IS1R | 182216..182865 | 649 | - | - | - |
| IScluster/Tn | ISKpn26 / ISKpn21 | 231047..236301 | 5254 | - | - | - |
| Integron | - | 43561..44259 | 698 | - | - | - |
| Integron | - | 49863..52778 | 2915 | dfrA12 / aadA2 | Folate&pathway&antagonist / Aminoglycoside | Trimethoprim / Spectinomycin, Streptomycin |

S 4 pGS32-1 conjugative transfer module prediction


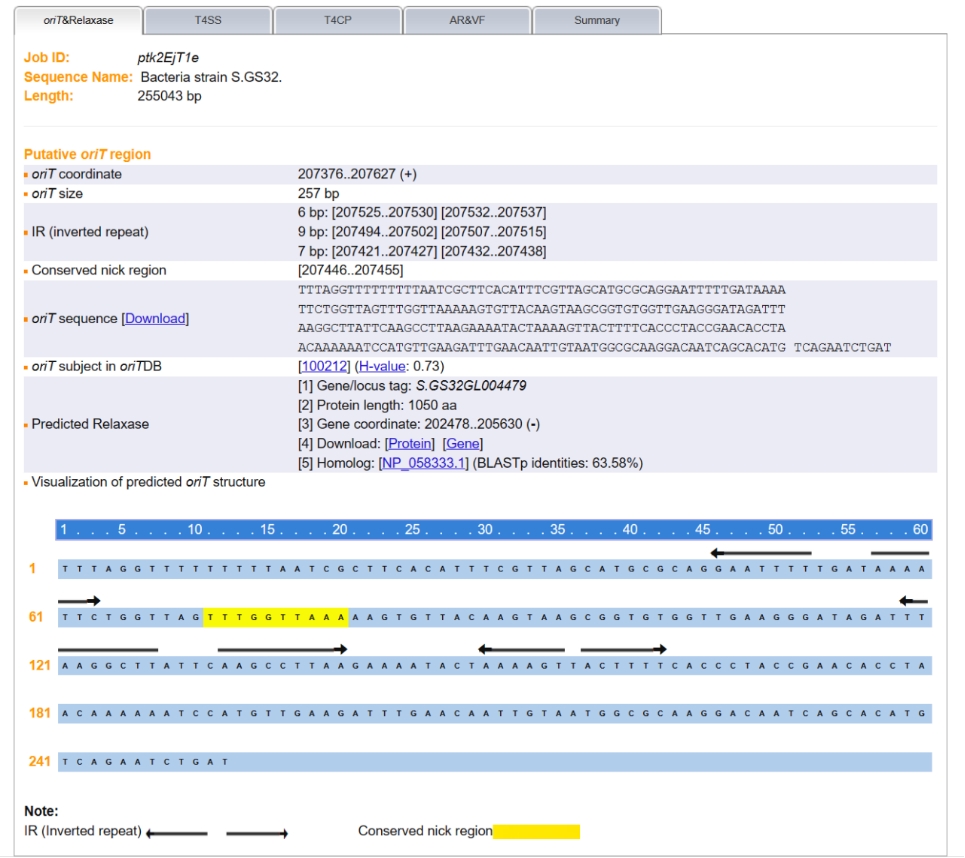

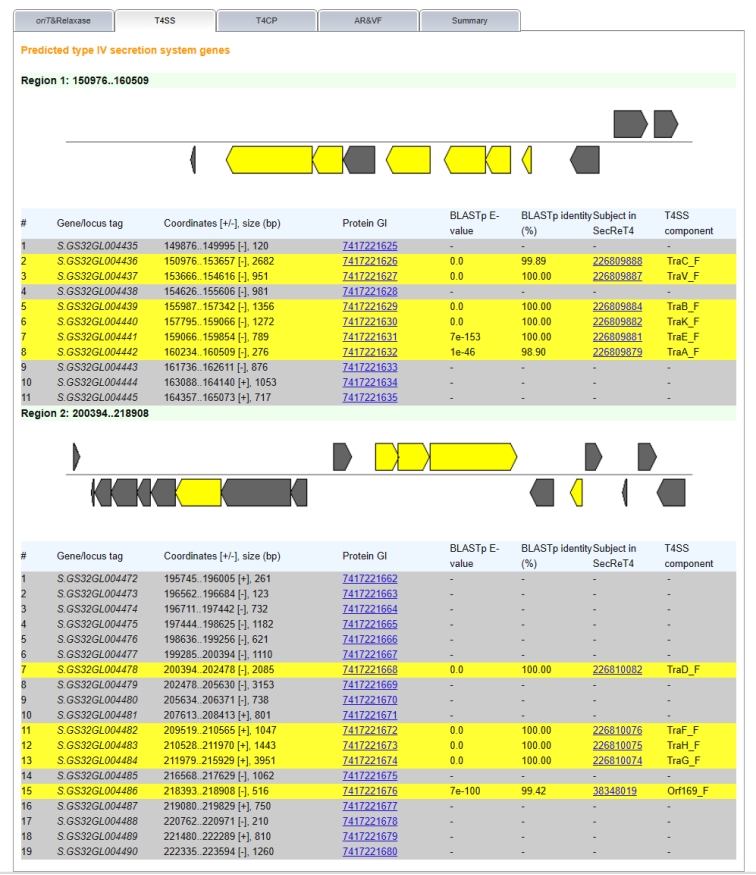
S 5 pGS32-1 carrying integron prediction

| **ID_integron** | **pos_beg** | **pos_end** | **strand** | **annotation** | **type** |
| --- | --- | --- | --- | --- | --- |
| integron_01 | 43422 | 43480 | -1 | attI_1 | In0 |
|  | 43505 | 43539 | 1 | Pint_1 | In0 |
|  | 43531 | 43557 | -1 | Pc_1 | In0 |
|  | 43561 | 44259 | 1 | intI | In0 |
| integron_02 | 49863 | 49922 | -1 | attC | complete |
|  | 49924 | 50703 | -1 | protein | complete |
|  | 50719 | 50778 | -1 | attC | complete |
|  | 51039 | 51128 | -1 | attC | complete |
|  | 51123 | 51620 | -1 | protein | complete |
|  | 51626 | 51684 | -1 | attI_1 | complete |
|  | 51709 | 51743 | 1 | Pint_1 | complete |
|  | 51735 | 51761 | -1 | Pc_1 | complete |
|  | 51765 | 52778 | 1 | intI | complete |

S 6 Comparative genomic reference sequence

| **ID** | **Sequence** | **Organism** | **Accession** |
| --- | --- | --- | --- |
| 1 | pCf.1_2 | *Citrobacter freundii* | NZ_OK649970 |
| 2 | pMCR-SCNJ07 | *Enterobacter hormaechei* | NZ_MK933279 |
| 3 | pSE15-SA01028 | *Salmonella enterica* subsp. *enterica* | NZ_CP026661 |
| 4 | pRH-R27 | *Salmonella enterica* subsp. *enterica serovar* Infantis | LN555650 |
| 5 | pEcl4-1 | *Enterobacter hormaechei* | NZ_CP047741 |
| 6 | p707804-NDM | *Leclercia adecarboxylata* | NZ_MH909331 |
| 7 | pN1863-HI2 | *Enterobacter cloacae* | NZ_MF344583 |
| 8 | pOYZ4 | *Salmonella sp.* | NZ_MN539018 |
| 9 | pSW37-267109 | *Salmonella enterica* subsp. *enterica serovar* Worthington | NZ_CP051274 |
| 10 | pSCLC9-2_3 | *Klebsiella pneumoniae* | NZ_CP113218 |
